# Narrative Review: Quantitative EEG in Disorders of Consciousness

**DOI:** 10.3390/brainsci11060697

**Published:** 2021-05-25

**Authors:** Betty Wutzl, Stefan M. Golaszewski, Kenji Leibnitz, Patrick B. Langthaler, Alexander B. Kunz, Stefan Leis, Kerstin Schwenker, Aljoscha Thomschewski, Jürgen Bergmann, Eugen Trinka

**Affiliations:** 1 Graduate School of Information Science and Technology, Osaka University, Suita 565-0871, Japan; b-wutzl@ist.osaka-u.ac.jp (B.W.); leibnitz@nict.go.jp (K.L.); 2 Symbiotic Intelligent Systems Research Center, Osaka University, Suita 565-0871, Japan; 3 Department of Neurology, Christian Doppler Medical Center, and Centre for Cognitive Neuroscience, Paracelsus Medical University, Affiliated Member of the European Reference Network EpiCARE, 5020 Salzburg, Austria; s.golaszewski@salk.at (S.M.G.); patrickbenjamin.langthaler@stud.sbg.ac.at (P.B.L.); a.kunz@salk.at (A.B.K.); s.leis@salk.at (S.L.); k.schwenker@salk.at (K.S.); a.thomschewski@salk.at (A.T.); J.Bergmann@salk.at (J.B.); 4 Karl Landsteiner Institute for Neurorehabilitation and Space Neurology, 5020 Salzburg, Austria; 5 Neuroscience Institute, Christian Doppler Medical Center, and Centre for Cognitive Neuroscience, Paracelsus Medical University, 5020 Salzburg, Austria; 6 Center for Information and Neural Networks, National Institute of Information and Communications Technology, Suita 565-0871, Japan; 7 Department of Mathematics, Paris Lodron University of Salzburg, 5020 Salzburg, Austria; 8 Team Biostatistics and Big Medical Data, IDA Lab Salzburg, Paracelsus Medical University, 5020 Salzburg, Austria; 9 Spinal Cord Injury and Tissue Regeneration Center Salzburg, Paracelsus Medical University, 5020 Salzburg, Austria

**Keywords:** unresponsive wakefulness syndrome, minimally conscious state, EEG, quantitative EEG, disorders of consciousness, diagnosis, prognosis

## Abstract

In this narrative review, we focus on the role of quantitative EEG technology in the diagnosis and prognosis of patients with unresponsive wakefulness syndrome and minimally conscious state. This paper is divided into two main parts, i.e., diagnosis and prognosis, each consisting of three subsections, namely, (i) resting-state EEG, including spectral power, functional connectivity, dynamic functional connectivity, graph theory, microstates and nonlinear measurements, (ii) sleep patterns, including rapid eye movement (REM) sleep, slow-wave sleep and sleep spindles and (iii) evoked potentials, including the P300, mismatch negativity, the N100, the N400 late positive component and others. Finally, we summarize our findings and conclude that QEEG is a useful tool when it comes to defining the diagnosis and prognosis of DOC patients.

## 1. Introduction

In recent years, neurological intensive care and medical care in general have improved substantially. Hence, more people are surviving severe brain injuries. Thus, the percentage of people dying from traumatic brain injuries, for example, decreased from 16% in 2000 to 11% in 2010 [1]. Nevertheless, not all people fully recover, and many patients remain in a prolonged coma, defined as a state of absence of arousal (eye opening) and awareness (non-reflexive behavior or command following) [2], in the acute state, and a substantial number (10–15% [3]) of survivors stay with disorders of consciousness (DOC). DOC describe a continuum of states with no arousal or awareness to states of full arousal and awareness. Patients with DOC are categorized as being in one of three main states: (i) coma, (ii) unresponsive wakefulness state (UWS) and (iii) minimally conscious state (MCS) [4]. Patients in UWS open their eyes spontaneously but are unresponsive to external stimuli or show just reflex movements, see, e.g., [5,6,7,8]. Patients with MCS, in contrast, show evidence of awareness of themselves or of their environment, though this awareness fluctuates [4]. MCS plus (MCS+) patients show high-level behavioral responses, namely, command following and intelligible verbalization, or non-functional communication. On the other hand, MCS minus (MCS−) patients only show low-level behavioral responses. These may include visual pursuit, the localization of noxious stimuli or appropriate crying or smiling when exposed to emotional stimuli [9]. Patients that regain consciousness after being in MCS are referred to as being in emerging MCS (EMCS), i.e., they are already able to functionally communicate and functionally use objects [10]. Moreover, on the upper boundary of MCS, there is severe neurocognitive disorder (SND). Those patients show evidence of arousal and awareness, i.e., consciousness, but have severe impairment to two or more cognitive sub-functions [11]. Another state which must not be confused with DOC is the so-called locked-in syndrome (LIS). In contrast to patients with severe disturbances of consciousness, patients with LIS are aware of themselves and their environment but are fully de-efferentiated, due to bilateral transection of pyramidal tracts at the level of pons or cerebral peduncles, leading to complete immobility except for vertical gaze [2]. Some studies included conscious subjects (CS), i.e., patients with brain injuries who are fully conscious, e.g., [12].

Over the years, various scales have been introduced to categorize DOC patients, see, e.g., [13] for a review. The Glasgow–Liège Scale (GLS), which was introduced in 1982, is a combination of the Glasgow Coma Scale (GCS) [14] and quantified analysis of brain stem reflexes [15]. Moreover, there is the Innsbruck Coma Sale (ICS), which is similar to the GCS and also has number of separate assessments which are added up to an aggregate score [16]. The Wessex Head Injury Matrix (WHIM) was developed for assessing and monitoring patients’ recovery after severe head injury [17]. The state-of-the-art scale to assess coma in the non-emergency setting today is the JFK Coma Recovery Scale-Revised (CRS-R) [18], which is based on the Disability Rating Scale (DRS) [19] and the Coma Recovery Scale (CRS) [20]. The Coma/Near Coma (CNC) Scale is, similar to the DRS, related to the patient’s status, course and outline, but also to the underlying electro-neuro-physiological dysfunction [21].

The American Academy of Neurology and the American Clinical Neurophysiology Society define quantitative EEG (QEEG) as: “… the mathematical processing of digitally recorded EEG in order to highlight specific waveform components, transform the EEG into a format or domain that elucidates relevant information, or associate numerical results with the EEG data for subsequent review or comparison” [22]. It involves the use of computers, and several measures, e.g., the power spectrum, can be derived from it. In this review, we will critically assess the role of QEEG in the diagnosis and prognosis of patients with DOC. We will focus on QEEG because it is, other than functional magnetic resonance imaging (fMRI) or positron emission tomography (PET), non-invasive, less expensive, widely applicable and a bedside measurement.

Behavioral tests are the gold standard for diagnosis and the following prognosis, even though they have limitations [23]. Hence, clinicians and neuroscientists often seek for additional tests to behavioral tests. Here, we review the current literature on diagnosis and prognosis of DOC patients and attempt to give the reader an overview of the parameters that can be extracted from EEG and their usefulness. Our focus lies on the diagnosis and prognosis of DOC patients, since those are the two most important factors for clinical use. Diagnosis is the first step in clinic decision making, on which the choice of treatment is based. The right treatment can just be found with the right diagnosis at hand. Prognosis is important for ethical reasons and especially of interest to the relatives of the patients. Hence, we provide an overview of the research over the last 20 years (2000–2020) of all the different parameters extracted from EEG recordings used for diagnosis and prognosis, and we summarize our findings in two tables at the end. Some existing reviews just focus on prognosis [24] or just present work conducted on resting-state EEG [25], [26]. Other reviews focus on BCI [27] or also EEG reactivity and transcranial magnetic stimulation (TMS)-EEG [28,29]. Another review on EEG and neuroimaging can be found, e.g., in [30]. Moreover, there are also some reviews focusing on interventions and therapy [31,32]. We did not include any of the other mentioned topics, except diagnosis and prognosis, because we intended to write a focused review with just the two most important aspects for clinical practice, and including all mentioned aspects would go beyond the scope of our discussion.

## 2. Methods

**Figure 1:**
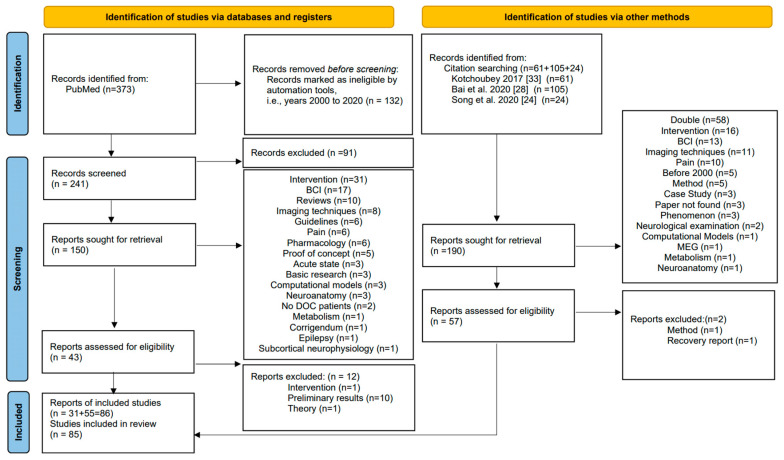
PRISMA flow chart (following the example in [42]) explaining our inclusion and exclusion criteria.

We searched the database PubMed for articles using the following search term: “(EEG OR electroencephalography OR electroencephalogram OR QEEG) AND (DOC OR disorders of consciousness OR vegetative state OR VS or unresponsive wakefulness syndrome OR UWS OR MCS OR minimally consciousness state OR coma)”, which returned over 10,000 results. Hence, we reduced our search to “EEG AND DOC”, which returned 214 results. Since we restricted our search, we checked the bibliography of all found papers for relevant studies and also included those in our research. After an initial screening of the titles and abstracts, we could reduce the number of papers before conducting a full-text analysis. We included 86 papers in this review (from our PubMed search and citation search from [24,28,33]). The criteria for study selection were as follows:

We focused on the diagnosis and prognosis of DOC patients using QEEG. Hence, we excluded papers focusing on basic research, e.g., [34], or on brain computer interfaces or treatment response, e.g., [28]. We did not include active paradigms such as the imaginary paradigm. Moreover, we excluded papers focusing on machine learning for which we refer the reader to [35]. Furthermore, we excluded papers dealing with other types of measurements, e.g., fMRI, PET or TMS. For a review about fMRI and QEEG for DOC patients, see, e.g., [30]. We also did not include the prediction of acute severe brain injury, where we refer to [36]. The entire process is summarized in the flow chart in Figure 1.

In order to be able to better compare the different results found in the literature, we performed a calculation of Cohen’s d [37] and its confidence interval for each of the investigated parameters whenever possible. Cohen’s d is a common way to measure the effect size of two subject groups, with values lying between 0 and 0.1 indicating no effect, values between 0.2 and 0.4 indicating a small effect, values between 0.5 and 0.7 indicating an intermediate effect and values larger than 0.8 indicating a large effect. Negative values describe an adverse effect [37]. Cohen’s d can be interpreted as the number of standard deviations that the means of two groups differ by.

We calculated Cohen’s d as follows:

1. If sample sizes (*n*_1_, *n*_2_), means (*mean*_1_, *mean*_2_) and standard deviations (*sd*_1_, *sd*_2_) were available, we calculated *d* directly as
  (1) $$d=\frac{mean_{1}-mean_{2}}{\sqrt{\frac{\left(n_{1}-1\right)*sd_{1}^{2}+\left(n_{2}-1\right)*sd_{2}^{2}}{n_{1}+n_{2}-2}}}$$
2. If a *t* statistic and sample sizes were available, we used
  (2) $$d=\sqrt{\frac{1}{n_{1}}+\frac{1}{n_{2}}}*t$$
3. For F statistics with the first degree of freedom equal to one, resulting from a comparison between two groups, we first calculated a *t* statistic by taking the square root of F and then proceeded as above.
4. For chi-squared statistics with one degree of freedom, we first transformed to a correlation via
  (3) $$r=\sqrt{\frac{\chi_{1}^{2}}{N}}$$
  and then to Cohen’s *d* via
  (4) $$d=\frac{2*r}{\sqrt{1-r^{2}}}$$
5. For an area under the curve (AUC), we used
  (5) $$d=\sqrt{2}*\Phi^{-1}\left(AUC\right)$$
  where Φ^−1^ is the inverse of the distribution function of the standard normal distribution.
6. For 2 × 2 contingency tables, we performed Fisher’s exact test as implemented in R using the command fisher.test. The odds ratios (OR) and limits of their confidence intervals were then transformed using
  (6) $$d=\frac{log\mathrm{OR}*\sqrt{3}}{\pi }$$
7. When confidence intervals were not given for the originally reported effect measure, we calculated confidence intervals for Cohen’s d using
  (7) $$lowerlimit=d-\Phi^{-1}\left(0.975\right)*\sqrt{\left(\frac{n_{1}+n_{2}}{n_{1}*n_{2}}+\frac{d^{2}}{2*\left(n_{1}+n_{2}-2\right)}\right)*\frac{n_{1}+n_{2}}{n_{1}+n_{2}-2}}$$
  (8) $$upperlimit=d+\Phi^{-1}\left(0.975\right)*\sqrt{\left(\frac{n_{1}+n_{2}}{n_{1}*n_{2}}+\frac{d^{2}}{2*\left(n_{1}+n_{2}-2\right)}\right)*\frac{n_{1}+n_{2}}{n_{1}+n_{2}-2}}$$

For actual calculation, we used either the statistical software package R (Version 4.0.5) [38], a website-based calculator [39] or a conversion table [40]. References for the above formulae can be found at [39], and the formula for the confidence interval can be found at [41], p. 238.

We summarized the results including the Cohen’s d values and confidence intervals in Section 4. We decided not to pool effects from different studies since the considerable heterogeneity in study designs and outcome measures meant they were largely not comparable with each other.

All abbreviation used throughout the paper can be found in Abbreviation.

## 3. Results

### 3.1. Diagnosis

#### 3.1.1. Resting-State EEG

Resting-state EEG is an easily applicable measurement where the subject is not required to perform any task, and where no stimuli are presented. Hence, it is ideal for DOC patients. The EEG oscillations are generally divided into the following bands: delta band (0.5–3 Hz), theta band (4–7 Hz), alpha band (8–13 Hz), beta band (14–30 Hz) and gamma band (>30 Hz). Sometimes, these bands are further divided into sub-bands, or authors use slightly different frequency boundaries. If this is the case, we will mention it when we describe the paper. An overview of the papers and a quick summary of the results of each study can be found in Table 1.

##### Spectral Power

Spectral power analysis is a standard QEEG method showing the distribution of the signal’s power over specific frequency bins or, in other words, the frequency content of the signal. The frequency bands that are most important for distinguishing patients in UWS, MCS and SND seem to be the alpha, delta and theta bands. The powers of delta and theta bands were increased in MCS when compared to SND patients [11]. Several studies showed that UWS patients had decreased alpha but increased delta power when compared to MCS patients [52,55,62,65,66]. Theta power is not so well studied, but it was found to be higher in UWS patients compared to healthy controls [55]. However, Piarulli et al. reported that UWS patients had lower theta power when compared to MCS patients [62], whereas another study presented no differences between UWS and MCS patients [65]. Hardly any papers deal with beta or gamma band frequencies. One study, however, showed no difference in beta and gamma band power between UWS and MCS patients [65]. Normalized delta power was found to be lower in patients with MCS than in patients with UWS. In contrast, normalized theta and alpha powers were higher in CS than in UWS patients [59]. A significant negative correlation between CRS values and relative delta power in the parieto-occipital, fronto-central and midline regions, as well as a positive correlation between CRS values and relative alpha power in the parieto-occipital, fronto-central and midline regions, was found [60]. The powers of delta and alpha bands were also found to have a correlation with CRS-R scores, i.e., alpha power increases and delta power decreases with increasing CRS-R values [56,65]. Moreover, Lechinger et al. found a positive correlation between the ratios of frequencies above and below 8 Hz and the CRS-R. Additionally, the spectral peak frequency was correlated with the CRS-R score of patients with UWS and MCS [55]. The theta band power was also found to significantly correlate with clinical variables such as the CRS-R score and other demographic factors [69]. When focusing on LIS patients vs. healthy controls, Babiloni and colleagues showed that alpha power was lower, whereas delta power was higher, in the LIS group [45]. Coleman et al. reported that the power ratio index, defined as the ratio of percentage power in slow-wave activity (delta and theta frequency bands) to that in fast-wave activity (alpha and beta frequency bands), was significantly higher in UWS compared to MCS patients. [73]. Source localization incorporating the low-resolution electromagnetic tomography (LORETA) model [74] was used in some studies, revealing higher amplitudes of theta frequencies and delta frequencies in posterior sources of MCS patients compared to SND patients. Furthermore, fast frequencies showed lower source magnitudes in MCS patients when considering the temporal and frontal lobes [11]. Naro et al. also used LORETA and found a significant difference in source power between UWS and MCS patients. Delta power is different in frontal sources, that is, it is increased in UWS. Theta power in frontal and parietal sources was more abnormal in UWS, as was alpha power in parietal and occipital sources. Beta power in central sources showed a positive correlation with the motor item score. Moreover, gamma power in parietal sources was also more abnormal in UWS [61]. Rossi Sebastiano et al. reported that the absolute total power is related to etiology. Thus, lower absolute total power was found in anoxic patients. However, the absolute total power was not able to distinguish between traumatic and vascular etiologies. Lutkenhoff et al. analyzed DOC patients, their power spectrum and subcortical damage measured via magnetic resonance imaging. They found that the EEG power spectra were associated with the subcortical damage of the patient’s brain. The ratio of beta to delta relative powers was lower with higher atrophy in the bilateral thalamus and globus pallidus. The power spectrum total density was lower with more widespread atrophy in the brainstem, the left globus pallidus and the right caudate [72].

##### Functional Connectivity

Functional connectivity describes different measures quantifying how neural activities of two different brain areas relate to each other. For a tutorial review, see [75]. Since there are many different types of measurements, it is hard to find multiple papers dealing with the same measurement. However, some publications focused on the number of connections in general. SND patients were found to have a larger number of connections than MCS patients [46,53]. Additionally, MCS patients had significantly higher connectivity in the alpha and beta bands when compared to UWS patients [52]. Coherence was also analyzed in some studies, but it was not possible to differentiate between MCS and UWS patients using coherence patterns [63]. Another research group, however, reported that coherence in alpha and beta frequencies was larger in UWS than in MCS patients [66]. Focusing on cross-approximate entropy, the interconnections of UWS patients were generally suppressed for local and distant cortical networks, whereas the interconnection of local cortical networks was increased for patients with MCS [50]. Granger causality is also a well-known parameter; however, no conclusive results were found when comparing SND and MCS patients [53]. The outgoing Granger causality distribution is wider in comparison to the incoming values for UWS, MCS and EMCS patients as well as healthy controls. Focusing on the UWS group, it was found that electrodes from central, occipital and temporal areas showed dissymmetry between outgoing and incoming information. Comparing MCS and EMCS patients, the bottleneck regions move more towards occipital areas. Moreover, considering healthy controls, lateral parietal electrodes showed the biggest difference between incoming and outgoing information. Differences in the distribution of the overall redundancy and synergy balance between all groups (UWS, MCS, EMCS, healthy controls), except EMCS vs. controls, were high [58]. Transfer entropy could not differentiate four groups, UWS, MCS, EMCS and healthy controls [58]. However, another study could distinguish between UWS and MCS patients using transfer entropy, yielding the best results in the alpha band [66]. Weighted symbolic mutual information increases with the level of consciousness and can be used to distinguish between UWS patients, MCS patients and conscious patients [54,66]. Moreover, this measure does not depend on etiology or the time since insult [54]. Höller et al. investigated 44 different biomarkers and found that partial coherence, generalized partial directed coherence and directed transfer function could differentiate UWS patients from MCS patients as well as healthy controls from UWS and MCS patients [57]. Considering weighted symbolic mutual information, it was found that inter-electrode information exchanges were higher in brain-injured but conscious patients when compared to UWS patients, and they were lower in the theta and alpha bands for UWS patients than for MCS and conscious patients [59]. Using symbolic transfer entropy, altered directed information flow was found for DOC patients. This indicates impaired feed-backward connectivity [64]. Focusing on the CRS-R, it was found that alpha band connectivity, both the imaginary and real parts of coherence, the phase lag index and quadratic self-coupling in different bands (delta, theta and alpha) were correlated with the CRS-R [34,52,69].

##### Dynamic Functional Connectivity

The above measures use static functional connectivity; however, there is also a dynamic approach—see, e.g., [76]. Time-dependent phase synchronization of delta, theta, alpha, beta and gamma bands was analyzed. The changes in dynamic functional connectivity matrices and the topography (mainly in the gamma range) over time were significantly different between MCS and UWS patients. Moreover, it was found that the degree of dynamic functional connectivity and the CRS-R were significantly correlated [65].

##### Graph Theory

Graph theory is a mathematical tool, which includes the use of nodes—for EEG, typically electrodes or sources—and connections, aspects of the signal in the node, between them. The combination of nodes and edges forms a network which can be characterized via different measures—see, e.g., [77,78]. Local and global efficiency were reduced, and fewer hubs were found in the alpha band of DOC patients’ networks when comparing them to healthy controls. Moreover, network modules in the alpha band of DOC patients were spatially circumscribed. Considering the delta and theta bands, the differences between the metrics were partially reversed, being more similar to each other in the DOC patient group than to the healthy subjects. Furthermore, metrics of network efficiency of the alpha band correlated with the level of behavioral awareness [56]. The clustering coefficient and the characteristic path length (of all networks from delta, theta, alpha and beta frequencies) were both able to distinguish between UWS and MCS patients [66]. Subnetworks in UWS patients have decreased functional connectivity compared to MCS patients. Considering nodes, altered functional topology of regions in the limbic and temporo-parieto-occipital parts was found in UWS patients [67]. DOC patients showed impaired network integration, i.e., global information processing, when compared to healthy controls. Moreover, network segregation, i.e., local information processing, was increased in DOC patients compared to healthy controls. The level of consciousness was lower when the large-scale functional brain networks’ integration was lower [68]. Cai et al. analyzed network segregation and integration in cross-frequency bands using a multiplex framework. Integration of the networks of the five common frequencies resulted in a frequency-based multiplex network. They found that networks of DOC patients have decreased segregation and increased integration when it comes to inter-frequency dynamics. Increased temporal and spatial variability were found to correlate with the level of consciousness. The behavioral performance of DOC patients was significantly correlated with the alteration of cross-frequency networks on a global as well as a local scale [70]. Using multiplex and multilayer network metrics, it was shown that the heterogeneity of functional networks, especially the fronto-parietal network, could discriminate between UWS and MCS patients. These results could not be found when focusing on individual frequency-specific networks. A positive correlation between the hub vulnerability of the regions and the behavioral performance was found. Considering multiplex analysis, a separation at the group level could be achieved. On the other hand, multilayer analysis was able to differentiate DOC patients individually [71].

##### Microstates

EEG microstates are stable scalp potential fields, which last a short time. We refer the reader to [79] for a review. Altered states of consciousness were related to a decreased number of microstate types. Moreover, unawareness and lower diversity in alpha-rhythmic microstates were associated. The duration and probability of the occurrence of fast alpha-rhythmic microstates were related to consciousness, whereas the duration and probability of occurrence of slow alpha-rhythmic, delta-rhythmic and theta-rhythmic microstates were related to unawareness [51]. The percentage of time which was spent in microstate D in the alpha frequency band was the best measure for classifying UWS and MCS patients [66].

##### Nonlinear Measures

Besides the above, there are a lot more measures which can be derived from transformed EEG signals, which will be summarized in this section. The bispectral index, a measurement coming from anesthesia monitoring, was found to have a correlation with the level of consciousness, measured via GLS and WHIM. A bispectral index cut-off of 50 was able to distinguish between unconscious (coma or UWS) patients and conscious (MCS or EMCS) patients [43,44]. The mean approximate entropy of UWS patients was lowest, followed by MCS patients and controls [47,49,50,66]. Mean EEG entropy values were found to be lower in UWS than in MCS patients [48]. The Lempel–Ziv complexity was highest for conscious patients (stroke or brain trauma), followed by MCS and UWS patients [12]. The Kolmogorov–Chaitin complexity increased with the state of consciousness and was successfully used to differentiate between UWS and MCS patients, especially when focusing on the parietal region. Permutation entropy-based measures could be used to differentiate UWS patients from others, especially in the theta range. A higher permutation entropy corresponded to a higher state of consciousness (most successful when derived from centro-posterior regions) [59,64,66]. MCS patients were found to have a higher mean spectral entropy than UWS patients [62].

#### 3.1.2. Sleep Patterns

In this section, we describe the findings from diagnosis using sleep patterns. See Table 2 for an overview. When it comes to rapid eye movement (REM) sleep, different results are found in the literature. Oksenberg et al. found that all UWS patients showed REM sleep [80], whereas another study reported that during the night, MCS patients, but not UWS patients, showed REM sleep stages [81]. Mertel et al. found that 12% of MCS patients, 44% of UWS patients and non-tetraplegic control patients lacked REM sleep patterns [82]. For UWS patients with REM sleep, it was shown that the duration of REM sleep is lower than for healthy subjects, but not if only focused on nocturnal periods [80]. Moreover, the density of REM was reduced in UWS patients when comparing to healthy subjects [80]. Non-REM 2 stages were found more often in MCS than in UWS patients [83].

Slow-wave sleep was found in 30% of UWS and 80% of MCS patients [84]. Rossi Sebastiano et al. reported that slow-wave sleep occurs more often in MCS than in UWS patients [83]. Another study, however, found no slow-wave sleep in UWS during the night at all. They also showed a homoeostatic decline in slow-wave activity in MCS patients [81]. Children with DOC had globally reduced slow-wave activity build-up when compared to healthy and brain-injured, but conscious, children [88]. Slow-wave sleep has a correlation with the CRS-R. The CRS-R was correlated with slow-wave activity [85]; however, it was negatively correlated with the amount of slow waves during the night period [89]. The duration of slow-wave sleep was positively correlated to the CRS-R [83].

Sleep spindles seem to be correlated with consciousness and several cognitive processes performed during the night (e.g., memory consolidation). As expected, it was found that 21% of MCS and 62% of UWS patients do not present with sleep spindles [82]. Additionally, the power of sleep spindles was lower in UWS than in MCS or EMCS patients [90]. Moreover, DOC patients, who showed command following in fMRI, had spindle activity during their sleep [87]. However, sleep spindles did not vary between day and night sleep in UWS and MCS patients. There was a correlation between the behavioral diagnosis (UWS, MCS and LIS) and sleep spindles [85], as well as the density of sleep spindles during night periods in the parietal areas for UWS, MCS and healthy controls [89].

Another study compared polysomnography and evoked potentials and found that the correlation with the clinical evaluation (including CRS-R, DRS and GCS) is higher than the one between evoked potentials and clinical evaluation [86].

#### 3.1.3. Evoked Potentials

Evoked potentials are *“electrical manifestations of the brain’s reception of and response to an external stimulus”* [91]. In the EEG, these evoked potentials can be seen as peaks, which can be either positive or negative. We use the conventions of naming the peaks P (positive) or N (negative) and provide the latency in milliseconds (ms). For more information on evoked potentials, see, e.g., [92].

Most papers that deal with evoked potentials use auditory stimuli. We focus here on late auditory evoked potentials, i.e., more than 50 ms post-stimulus. The early auditory evoked potentials occur around 2–8 ms post-stimulus and reflect activity from the auditory pathway, whereas the late auditory potentials are a sign of cognitive processing [93]. For papers using other stimuli, we will specifically mention them in the following. Table 3 shows an overview and a summary of all studies presented in this section.

##### P300

The P300 is evoked by oddball paradigms. This means that a series of similar stimuli is presented to the subject which is suddenly interrupted by a different stimulus. This can be, for example, a different tone in a series of similar tones, or one’s own name in a series of names. It is often either passive (just by listening for auditory stimuli) or active (by counting the odd stimulus). The P300 was found to be different among subgroups of DOC patients. As such, the P300 was found in healthy, LIS and MCS patients, but not in most UWS patients [96,109,121]. Another study also reported moderate differences in the P300 in different patient groups (UWS or MCS) [59]. Kempney et al. found statistically significant differences in EEG responses to the patients’ (UWS/MCS) own name, which were sometimes similar to those of healthy controls [118]. Sergent and co-authors introduced a 1.5-h EEG protocol and found a significant P300 in 9 out of 15 healthy subjects, 1 out of 4 UWS and 4 out of 8 MCS patients and none in the only conscious patient [116]. Comparing the passive to the active paradigm also revealed good results in the sense that MCS patients and healthy controls showed a larger P300 in the active vs. the passive paradigm. This could not be observed for UWS patients [97,105]. Another study reported higher responses in the counting task (active) than in the passive task for all MCS and healthy subjects at an individual level [108]. Real at al. found that the P300 was lower in patients (UWS and MCS) when compared to healthy controls but could not differentiate between the two patient groups [114]. Schnakers et al. also tried to find differences between MCS+ and MCS− in their study which also included UWS and healthy controls. Here, 5 out of 8 MCS+, 3 out of 8 MCS− and 1 out of 10 UWS patients showed an enhanced P300 amplitude when comparing the active to the passive condition. No difference between MCS+ and healthy controls was found [111]. What was also considered was the latency. Hence, it was found that the P300 latency is significantly delayed in patients (UWS and MCS) when compared to healthy subjects [96,116]. Another study even found a larger increase in latency for UWS patients when compared to MCS patients and healthy controls [117]. Cavinato et al. found a correlation between the level of complexity of the stimulus and the increase in P300 latency for MCS patients and healthy controls, but not for UWS patients [101]. Considering the etiology, the novelty P300 was less often found in patients with anoxia than those with any other etiology [99], and Wu et al. showed that the P300 was clearer in non-traumatic patients when compared to patients with traumatic etiologies [121]. Annen and colleagues investigated the P300 responses to auditory and vibrotactile stimulation. They did not find differences in the P300 characteristics when comparing UWS to MCS patients or even when comparing traumatic vs. non-traumatic etiologies. The performances of the two different stimuli (auditory and vibrotactile) were independent of each other [120]. Investigating the P300 response in relation to the CRS-R score, it was shown that the amplitude of the novelty P300 is correlated with the CRS-R and even more with the auditory sub-score [105]. Gibson et al. focused on the P300a and P300b, and they reported that 8 out of 13 patients showed the P300a, but not the P300b. Only patients who were able to follow commands were also found to have event-related potentials of attentional orienting [113]. Another study even found one UWS patient who presented the P300a as well as the P300b [104].

##### Mismatch Negativity (MMN)

The mismatch negativity (MMN) is a negative component which is found as a response of the brain to the violation of a rule. This can be a sequence of tones which is interrupted by another tone but can be also designed for basically any other sequence of stimuli. For more details and underlying mechanisms, see [122]. When it comes to MMN and DOC patients, different results have been published. One study reported that MMN was found in 5 out of 27 patients (UWS and MCS) [99], and another reported it in 7 out of 12 [98], while another study found MMN in all UWS and MCS patients [105]. Erlbeck et al. found MMN in just 2 out of 19 DOC patients [115]. Some studies tried to distinguish UWS and MCS patients using MMN. It was found that the presence of MMN does not differentiate the two groups, but its significance was lower in UWS patients [102]. Another study was able to distinguish healthy from DOC patients but not the two patient groups (UWS, MCS) [59]. Wang et al. reported higher MMN latency for UWS patients when compared to MCS patients or healthy controls [117]. A relation between the CRS and MMN was found, i.e., the amplitude was higher for higher levels of consciousness [102]. Boly et al. found that effective connectivity during MMN revealed impaired backward connectivity from the frontal to temporal areas in UWS [100].

##### N100

The N100 belongs to the negative evoked potentials and has its peak at around 100 ms after the stimulus. It is largest in central areas but can be found at many sites [93]. Kotchoubey et al. reported that more frequent N100 components of event-related brain responses to stimuli of different complexity levels were related to a lower level of disability. The N100 was found more frequently in MCS than in UWS patients [95]. Wu et al. observed an N100 response to auditory stimuli in both MCS and UWS patients [121].

##### N400

The next peak we want to focus on is the negative peak at around 400 ms, i.e., the N400, which was found to have high effectiveness when it comes to examining aspects of language processing [123]. All DOC patients showed a higher N400 peak amplitude in the fronto-central regions as an answer to incongruous words or sentences [103,107]. UWS patients were found to have a delayed N400 in incongruous conditions when comparing them to MCS patients. Moreover, there was a correlation between the clinical scales (CNC and DRS) and the peak amplitude as well as latency [107]. The N400 was observed in the UWS group, the MCS group and healthy controls [110]. Beukema et al. found cortical responses to sound in all patients. In some patients, the auditory processing level exceeded what was expected from behavioral assessment. However, auditory processing did not differentiate between UWS patients and MCS patients [112]. Another study reported that most DOC patients did not show any responses [115]. However, Schoenle and Witzke could use the N400 to distinguish non-UWS, near-UWS and UWS [94].

##### Late Positive Component (LPC)

The LPC is found over parietal brain parts and in the interval of 400–800 ms post-stimulus and is important in memory paradigms [124]. Rohaut et al. observed that LPC was present in just 6 out of 19 healthy controls, 5 out of 14 MCS patients and in just one out of 15 UWS patients [110]. Another study, not distinguishing UWS and MCS, found an LPC in 2 out of 19 DOC patients [115]. Wu et al. found no LPC in UWS [121].

##### Other Measures

The P200 occurs more frequently in MCS compared to UWS [95]. Considering healthy controls, a large global effect on the global field power plots was found. This could not be statistically significantly observed for the other groups, i.e., conscious patients, UWS and MCS patients. However, an analysis could confirm a relationship between the CRS and the presence of a global effect [102]. The response score to visual stimuli increased with increasing consciousness over time. Visually evoked potentials were smaller in amplitude and longer in latencies when comparing UWS patients to healthy controls [106]. Contingent negative variation was significant in all healthy controls and a conscious patient, for five out of eight MCS and for three out of four UWS patients. Moreover, action anticipation, attention shift to the cue side and significant contextual modulation did not provide any significant results. Furthermore, local incongruence detection and lateralized readiness potential were more often observed in healthy controls, followed by conscious patients, MCS patients and UWS patients [116]. DOC patients were found to have a lower number of trials with delta event-related synchronization. Moreover, a positive correlation between the P300 and the number of epochs with delta event-related synchronization was observed [119].

### 3.2. Prognosis

The literature on prognosis is very heterogeneous concerning the follow-up time, and the number of papers is less than that for diagnosis. In the following, we summarize the most important results.

#### 3.2.1. Resting State

An overview of this section is given in Table 4.

##### Spectral Power

Alpha power seems to be an indicator for a good outcome. Hence, a correlation between alpha power and recovery of UWS patients after three months was found [125]. Moreover, the power of alpha performed even better at predicting the outcome (follow-up 589.26 ± 1125.32 days) than indexing consciousness [66]. A bad outcome (after six months) was associated with a higher probability of slow theta and delta oscillations, in combination but also on their own. Furthermore, patients who survived had a higher probability of alpha and fast theta oscillations, again in combination or on their own [126]. It was found that the theta band was important, that is, the higher the values of the normalized power, the higher the chance of recovery (<42 days) [59]. Furthermore, the power of delta frequencies also performed better at predicting the outcome (follow-up 589.26 ± 1125.32 days) than indexing consciousness [66]. Bareham et al. were able to predict the CRS-R of the follow-up measurement (three months later) by the present EEG recordings [69].

##### Functional Connectivity

The strength as well as the number of functional connections was statistically higher in the first assessment (three months post-injury) for patients who recovered (three months later) in comparison to patients who did not recover [127]. Parietal and fronto-parietal coherence could predict recovery from UWS to MCS in a follow-up measurement 12 months later [63]. Moreover, it was shown that coherences for all frequencies were higher for patients with an improved outcome (follow-up 589.26 ± 1125.32 days). Focusing on the imaginary part of coherence, only the beta band could reach significant results [66]. Another study showed that delta frequency network centrality could predict the outcome after one year [128]. Moreover, weighted symbolic mutual information and transfer entropy were also effective measures for predicting the outcome (follow-up 589.26 ± 1125.32 days) [66]. Frontal quadratic phase self-coupling in the theta band significantly differentiated between patients who recovered and those who did not (follow-up three months) [34].

##### Graph Theory

Non-traumatic patients who had a positive outcome (after one year) showed significantly higher mesoscale modularity within the delta band, while traumatic patients showed significantly higher microscale clustering coefficients for networks of the delta frequency [128]. The average clustering coefficient calculated from thresholding alpha and beta coherences predicted the outcome for a follow-up after evaluation of 589.26 ± 1125.32 days (the thresholding did not have much effect). The clustering coefficient in the theta range also showed significant results, whereas the path length failed to provide significant results [66]. A lower clustering coefficient, as well as a higher path length variance and modularity, was reported for patients with a favorable outcome (three months later) compared to those with an unfavorable outcome, at a group level. Considering all features, the variance of the path length had the best positive predictive value for a favorable outcome as well as specificity for an unfavorable outcome [129].

##### Microstates

Microstate A, the first of the four global microstates, was the most informative one for prediction (follow-up after 589.26 ± 1125.32 days), i.e., the duration of this state in the delta band, the frequency and percentage of time spent in this state in the theta band and the frequency of the microstate in the band from 2 to 20 Hz were all significant [66].

##### Nonlinear Measures

Patients who recovered (one year post-insult) had higher bispectral indices than those who did not [44]. UWS patients who had the lowest approximate entropy values remained in UWS or died (6 months later). On the other hand, patients with the highest values of approximate entropy became MCS patients or even better [49]. Approximate entropy in the alpha band was successful when predicting the outcome (follow-up after 589.26 ± 1125.32 days) but performed worse than permutation entropy in the delta and theta bands [66].

#### 3.2.2. Sleep Patterns

Sleep patterns seem to be a good indicator for recovery—see Table 5 for an overview. The appearance of organized sleep patterns predicted a positive outcome (3–34 months later) [130]. A better outcome (follow-up 18.5 ± 9.9 months) was correlated with the visual index indication of sleep integrity, and adding a quantitative sleep index further empowered the prediction [131]. Another study also reported a significant correlation between the consciousness state (conscious including MCS+, MCS−, EMCS and conscious without DOC, vs. non-conscious, including UWS and death) after one month for patients initially in a coma and the on-admission sleep EEG patterns [132]. Focusing just on REM sleep, no significant differences between UWS patients who recovered after 6 months and those who did not were found [80]. The presence of sleep spindles is related to a clinical improvement after 6 months [84]. Parietal sleep spindles were even linearly correlated with the outcome (1–150 months) [89]. Parietal slow-wave activity build-up was lowest in children who had a poor outcome (time interval between 16.1 and 1.5 months) [88].

#### 3.2.3. Evoked Potentials

The results of the papers dealing with prognosis and evoked potentials can be found in Table 6. In one study, all but one patient who showed a parietal component in the late part of the P300 woke up (defined as good recovery, moderate or severe disability) three months after coma onset [133]. A detectable P300 was found more often in post-traumatic UWS patients who regained consciousness one year later compared to those who did not [134]. However, another paper reported that a P300 was found in many UWS and MCS patients, but it was not correlated with outcome (2–14 years) [135]. Patients with a two-peak P300 to the oddball own name paradigm had a higher chance of recovering within a short time [109]. Another study found no correlation with the outcome (6 months post-injury) and EEG background activity or the P300 to event-related potentials [136]. MMN was related to a better outcome (6 months later) [95] and recovery of consciousness (three months later) [98]. A significant relationship between speech N400 occurrence and recovery (2–14 years) was found [135]. Faugeras and colleagues used the protocol by Bekinschtein et al. [137]. They found two UWS patients who showed a neural signature of consciousness by the given protocol. It was also these two patients who showed clinical signs of consciousness after three to four days [138]. In a re-analysis focusing on the global field power, only UWS patients presenting with a global effect showed improved consciousness after the EEG measurement (within three and four days) [102]. Wijnen et al. investigated UWS patients who were regaining consciousness using visual stimuli. They also assessed the long-term outcome (two to three years later) and found that visual evoked potentials from the first measurement were related to the long-term outcome [106]. Xu et al. reported that somatosensory evoked potentials were correlated with outcome (one year later) [139].

## 4. Conclusions and Discussion

### 4.1. Diagnosis

Many studies investigated the power spectra of UWS and MCS patients. The conclusion of these papers is that the delta power and the power ratio index are lower in MCS when compared to UWS, while the alpha power is higher in MCS patients than in UWS patients. The connectivity measures reveal that connectivity, dynamic functional connectivity, the imaginary part of coherence, phase lag entropy, the phase lag index, quadratic self-coupling in the alpha band, transfer entropy and weighted symbolic mutual information are all higher with a higher level of consciousness. Coherence, the phase locking index in the delta band and quadratic self-coupling in the theta band were lower for MCS than for UWS patients. The graph theory part showed a higher clustering coefficient, hub vulnerability, network integration and local and global efficiency in the alpha band, but a lower characteristic path length and network segregation for a higher consciousness level. Furthermore, more microstates are correlated with higher consciousness. From the nonlinear dynamics section, we learn that the bispectral index, approximate entropy, entropy, Lempel–Ziv complexity, Kolmogorov–Chaitin complexity and permutation entropy are all higher in MCS than in UWS patients. The section about sleep in DOC patients (Section 3.1.2) shows us that REM duration, sleep spindle occurrence and spindle power are increased with increased consciousness, i.e., these parameters are higher in MCS than in UWS patients. The last diagnostic section about evoked potentials (Section 3.1.3) lets us conclude that the N100, N400, P200, P300, MMN, LPC, global effect and visual evoked potentials have greater values for MCS compared to UWS patients. Some measures, e.g., slow-wave sleep and theta power, have been found to be higher with higher consciousness in some studies, whereas the opposite was reported in other papers. For a full summary of the different parameters and the diagnostic values including references, see Table 7.

### 4.2. Prognosis

Considering prognosis, the consensus is that higher values indicate a better prognosis for nearly all measurements of the alpha power, approximate entropy, bispectral index, coherence, entropy, global effect, mesoscale modularity in the delta band, microscale clustering coefficient in the delta band, MMN, modified Valente‘s grade, modularity, N400, number of functional connections, organized sleep patterns, P300, partial coherence, quadratic phase self-coupling in the theta band, sleep spindles, slow-wave build-up, strength of functional connections, theta normalized power, fast theta power, transfer entropy, variance of path length, visual evoked potentials and weighted symbolic mutual information in the alpha band. The only parameters which are higher for worse outcome are characteristic path length, delta power, network centrality in the delta band and slow theta power. The results considering the clustering coefficient were mixed. Table 8 shows an overview of all parameters for prognosis with their respective references.

### 4.3. Conclusions

In this narrative review, we discussed different metrics that can be extracted from EEG. However, not all of these measures have been equally well investigated and, thus, not all of them can be immediately clinically applied. The measures that are most often reported are alpha and delta power. These are easy to calculate and seem to provide conclusive results and as such should be brought into clinical practice. The theta band, even though often reported, does not seem to provide any conclusive results and as such should be investigated in further studies before bringing it into clinical practice. Sleep EEG is often used and reveals consistent results. The P300 is another measure that should be used in clinical practice because several papers are published on this topic with matching results.

What also has to be kept in mind is that the sample size of the different papers varies a lot. This means that not all results are equally trustworthy. The results of studies which include more subjects and have smaller confidence intervals for their parameters can be seen as more reliable, whereas studies with small sample sizes or large confidence intervals need to be treated with caution.

Concluding our review, we can say that the diagnosis and prognosis of DOC patients are still very difficult tasks. However, QEEG, especially resting-state analysis and sleep patterns, should become a part of the daily routine when treating these patients because it is easy to measure and provides conclusive results.

## Figures and Tables

**Table 1:** Overview of findings for diagnosis and resting-state EEG; abbreviations can be found in Abbreviation.

Authors and Reference	Patient Sample	Finding
Schnakers et al. [43]	11 Coma 32 UWS 42 MCS 21 EMCS	Nonlinear measures bispectral index highest correlation with the level of consciousness (via GLS and WHIM)
Leon-Carrion et al. [11]	7 MCS 9 SND	Spectral power EEG power spectra different in MCS and SND, MCS increased power compared to SND higher amplitudes of theta and delta frequencies in posterior sources of MCS compared to SND fast frequencies showed lower source magnitudes in the temporal and frontal lobes in MCS compared to SND
Schnakers et al. [44]	16 Coma 13 UWS 30 MCS 13 EMCS	Nonlinear measures bispectral index had the highest correlation with behavioral scales when comparing to other parameters, the only parameter which was able to disentangle UWS and MCS
Babiloni et al. [45]	13 LIS 15 HC	Spectral power power of alpha 2 (individual alpha frequency −2 Hz to individual alpha frequency) and alpha 3 (individual alpha frequency to individual alpha frequency +2 Hz) lower in LIS compared to HC power of delta sources in temporal, central, parietal and temporal regions was higher in LIS compared to HC
Pollonini et al. [46]	7 MCS 9 SND	Functional connectivity SND larger number of connections than MCS in all frequency bands significant difference in the number of connections between parieto-occipital and temporal areas in the delta band when comparing MCS and SND significant difference for the frontal area input from all other cortical areas in the beta band
Sarà and Pistoia [47]	10 UWS 10 HC	Nonlinear measures mean approximate entropy of UWS was lower than in HC
Gosseries et al. [48]	6 Coma 24 UWS 26 MCS	Nonlinear measures mean entropy values lower in UWS compared to MCS entropy cut-off of 52 could differentiate acute (≤1 month post-injury) unconscious patients from MCS with a specificity of 90% and a sensitivity of 89%, whereas in chronic (>1 month post-injury) patients, the entropy measurements did not give any reliable diagnosis
Sarà et al. [49]	38 UWS 40 HC	Nonlinear measures mean approximate entropy is lower in UWS compared to HC
Wu et al. [12]	21 UWS 16 MCS 30 CS	Nonlinear measures approximate entropy, and Lempel–Ziv: CS had the highest nonlinear indices followed by MCS and UWS
Wu et al. [50]	30 UWS 20 MCS 30 CS	Functional connectivity interconnections of UWS generally suppressed for local and distant cortical networks interconnection of local cortical networks improved for MCS patients
Fingelkurts et al. [51]	21 UWS 16 MCS 5 HC	Microstates altered states of consciousness related to a decreased number of microstate types unawareness and lower diversity in alpha-rhythmic microstates also associated duration and probability for the occurrence of fast alpha-rhythmic microstates related to consciousness, duration and probability of occurrence of slow alpha-, delta- and theta-rhythmic microstates were related to unawareness
Lehembre et al. [52]	10 UWS 21 MCS	Spectral power UWS decreased alpha but increased delta power compared to MCS connectivity in the alpha and delta bands of UWS significantly lower than in MCS Functional connectivity imaginary part of coherence, coherence and the phase lag index: correlation between these measures and the CRS-R MCS significantly higher connectivity in alpha and theta band when compared to UWS
Leon-Carrion et al. [53]	7 MCS 9 SND	Functional connectivity higher number of functional connections between frontal and left temporal, frontal and parietal occipital and parietal occipital and left temporal regions in SND compared to MCS Granger causality, no conclusive results SND more connections than MCS, most pronounced in the delta, alpha and beta bands
King et al. [54]	75 VS 68 MCS 24 CS	Functional connectivity weighted symbolic mutual information increased with the level of consciousness and able to distinguish between UWS, MCS and CS, not depending on etiology or time since insult
Lechinger et al. [55]	8 UWS 9 MCS 14 HC	Spectral power spectral peak frequency correlated with the CRS-R UWS showed decreased alpha and increased delta and theta values compared to HC MCS patients no differences in frequency range when compared HC
Chennu et al. [56]	13 UWS 19 MCS 26 HC	Spectral power negative correlation between delta power and CRS-R positive correlation between alpha power and CRS-R Functional connectivity debiased weighted phase lag index no significant correlation in any frequency band Graph theory local and global efficiency reduced and fewer hubs in the alpha band of patients’ networks using modular span: network modules in the alpha band of DOC patients were spatially circumscribed, lacking the long-distance interactions structure of healthy subjects delta and theta band, the differences between metrics were partially reversed being more similar to each other in the patient group than to the subjects of the HC metrics of network efficiency of the alpha band correlated with the level of behavioral awareness
Höller et al. [57]	27 UWS 22 MCS 23 HC	Functional connectivity 44 different biomarkers, partial coherence, generalized partial directed coherence and directed transfer function distinguish UWS and MCS as well as HC from patients
Marinazzo et al. [58]	11 UWS 10 MCS 5 EMCS 10 HC	Functional connectivity outgoing Granger causality distribution is wider for all groups in comparison to the incoming values UWS: electrodes from central, occipital and temporal areas show dissymmetry between outgoing and incoming information comparing MCS and EMCS patients: the bottleneck regions move towards more occipital areas HC lateral parietal electrodes biggest difference between incoming and outgoing information transfer entropy cannot differentiate the four groups
Sitt et al. [59]	75 UWS 68 MCS 24 CS 14 HC	Spectral power normalized delta power decrease from UWS to MCS, successful separating UWS from non-UWS patients normalized theta and alpha power increased in CS compared to UWS increased power in parietal regions for theta and alpha frequency bands, differentiate UWS from non-UWS Functional connectivity phase locking index in the delta band weighted symbolic mutual information, inter-electrode information exchanges higher in CS when compared to UWS, in the theta and alpha band lower in UWS than in MCS and CS Nonlinear measures Kolmogorov–Chaitin complexity increased with state of consciousness, successfully differentiates UWS and MCS, especially parietal region permutation entropy-based measures could be used to differentiate UWS patients form others, especially theta range higher permutation entropy corresponded to a higher state of consciousness, especially centro-posterior regions
Rossi Sebastiano et al. [60]	85 UWS 57 MCS	Spectral power absolute total power not related to DOC classes but to etiology, i.e., significantly lower in anoxic patients but does not differentiate patients with traumatic or vascular etiologies UWS higher delta relative power in the fronto-central and parieto-occipital areas when compared to MCS significant correlation between CRS values and delta relative power in the parieto-occipital, fronto-central and midline regions significant correlation between CRS values and alpha relative power in the parieto-occipital, fronto-central and midline regions
Naro et al. [61]	6 UWS 7 MCS 10 HC	Spectral power UWS significant differences in the source power (of delta in frontal sources, theta in frontal and parietal sources, of alpha in parietal and occipital sources, of beta in central and gamma in parietal sources) alpha band most significant correlation with the level of consciousness central beta peaks correlate with motor ability dissociation between gamma and theta bands in parietal regions
Piarulli et al. [62]	6 UWS 6 MCS	Spectral power UWS lower theta and alpha power, but increased delta power compared to MCS Nonlinear measures MCS have higher mean spectral entropy than UWS MCS periodicity of spectral fluctuations of around 70 min (range 57–80 min) similar to values of healthy subjects, no periodicity in UWS spectral fluctuations
Schorr et al. [63]	58 UWS 15 MCS 24 HC	Spectral power EEG power over several areas, i.e., frontal, temporal, parietal and occipital do not distinguish UWS and MCS Functional connectivity frontal and parietal as well as fronto-parietal, fronto-occipital and fronto-temporal coherence: using those patterns not possible to differentiate UWS from MCS
Thul et al. [64]	UWS 8 MCS 7 HC 24	Functional connectivity symbolic transfer entropy: altered directed information flow for patients, indicates impaired feed-backward connectivity Nonlinear measures permutation entropy in patients has reduced local information content, this was most pronounced in UWS
Naro et al. [65]	17 UWS 15 MCS	Spectral power relative power of delta and alpha bands could differentiate UWS from MCS UWS nearly 80% of spectral power (overall) was within the delta band MCS alpha power twice as high as UWS power of theta, beta and gamma bands does not separate UWS from MCS delta power decreased with the CRS-R value and the alpha power increased with increasing CRS-R value Dynamic functional connectivity time-dependent phase synchronization of delta, theta, alpha, beta and gamma band, changes in dynamic functional connectivity matrices and the topography (mainly in the gamma range) over time differentiates MCS from UWS degree of dynamic functional connectivity and the CRS-R significantly correlated
Stefan et al. [66]	51 UWS 11 MCS	Spectral power alpha frequency power higher in MCS compared to UWS, delta frequency power was lower in MCS than UWS Functional connectivity coherence in alpha as well as beta frequencies greater in UWS weighted symbolic mutual information also significant at distinguishing UWS from MCS, namely, in the theta, delta and alpha range transfer entropy best results for the alpha band Graph theory clustering coefficient and characteristic path length (of all networks from delta, theta, alpha and beta frequencies) distinguish between UWS and MCS Microstates percentage of time spent in microstate D in the alpha frequency band was the best measure for classifying UWS and MCS Nonlinear measures approximate entropy higher in all frequency ranges for MCS compared to UWS permutation entropy significantly higher in alpha range in MCS than in UWS
Bai et al. [34]	31 UWS 20 MCS 20 HC	Functional connectivity correlation between quadratic self-coupling in different bands, i.e., delta, theta and alpha, and the CRS-R when using quadratic self-coupling in the theta band, differentiate between UWS, MCS and HC UWS patients higher quadratic self-coupling in the theta band on the left and a lower quadratic self-coupling in the alpha band in the right frontal regions, when compared to MCS
Cacciola et al. [67]	12 UWS 13 MCS	Graph theory network-based statistical analysis to find subnetworks in UWS (compared to MCS) decreased functional connectivity, mainly in the interhemispheric fronto-parietal connectivity nodes: altered functional topology of regions in the limbic and temporo-parieto-occipital parts in UWS
Rizkallah et al. [68]	9 UWS 17 MCS− 29 MCS+ 6 EMCS 21 HC	Graph theory DOC patients exhibit impaired network integration, i.e., global information processing network segregation, i.e., local information processing, increased in DOC patients compared to HC level of consciousness was lower when the large-scale functional brain networks’ integration was lower
Bareham et al. [69]	16 UWS 15 MCS− 7 MCS+ 1 EMCS	Spectral power relationship between alpha band connectivity and the clinical variable (CRS-R and demographic variable) Functional Connectivity theta band power significantly correlated to the clinical variables (CRS-R and demographic variable)
Cai et al. [70]	35 UWS 19 MCS 23 HC	Graph theory networks of DOC patients decreased segregation and increased integration when it comes to inter-frequency dynamics increased temporal and spatial variability correlates with the level of consciousness behavioral performance of DOC patients significantly correlates with the alteration of cross-frequency networks on a global as well as a local scale
Naro et al. [71]	17 UWS 15 MCS	Graph theory heterogeneity of functional networks, especially fronto-parietal, discriminate between UWS and MCS, but not when focusing on individual frequency-specific networks positive correlation between the hub vulnerability of the regions and the behavioral performance considering multiplex analysis, a separation at group level could be achieved multilayer analysis able to differentiate DOC patients individually
Lutkenhoff et al. [72]	37 UWS 17 MCS− 7 MCS+	Spectral power power spectra associated with the subcortical damage of the patient’s brain ratio of beta to delta relative power lower with higher atrophy in bilateral thalamus and globus pallidus power spectrum total density lower with more widespread atrophy in the brainstem, the left globus pallidus and the right caudate

**Table 2:** Overview of findings for diagnosis and sleep patterns; abbreviations can be found in Abbreviation.

Authors and Reference	Patient Sample	Finding
Oksenberg et al. [80]	11 UWS 6 HC	UWS patients have REM sleep periods REM sleep periods’ duration was significantly lower in UWS when compared to HC, but not if only focused on nocturnal periods chin twitches, leg muscle twitches and density of REM were significantly reduced in UWS compared to HC sawtooth waves lower, but not significantly, in UWS
Landsness et al. [81]	5 UWS 6 MCS	MCS: clear EEG changes which correlate with decrease behavioral vigilance all MCS patients alternating REM/non-REM sleep patterns all MCS homoeostatic decline in activity of slow waves through the night UWS behavioral sleep, but EEG patterns were unchanged between eyes open and muscle activity vs. eyes closed during the nighttime: UWS patients do not show slow wave sleep or REM sleep stages, no homoeostatic regulation of slow-wave activity
Cologan et al. [84]	10 UWS 10 MCS	sleep–wake cycles in 3 UWS and 5 MCS patients slow-wave sleep in 3 UWS and 8 MCS
Malinowska et al. [85]	11 UWS 20 MCS 1 LIS 5 HC	CRS-R correlated with appearance of EEG sleep patterns with sleep spindles, deep/light sleep cycles and slow-wave activity behavioral diagnosis correlated with the appearance and variability over time of the different frequency rhythms, i.e., alpha, beta and theta using EEG profiles, UWS and MCS correctly classified (87%)
de Biase et al. [86]	27 UWS 5 MCS	polysomnography better correlation with CRS-R, GCS and DRS than evoked potentials
Forgacs et al. [87]	8 UWS 23 MCS 13 EMCS	DOC patients, who showed evidence of command following in fMRI, have well-organized EEG background during their wakeful times and spindle activity during their sleep periods
Mouthon et al. [88]	4 MCS 1 EMCS 5 CS 10 HC	children with DOC globally reduced slow-wave activity build-up, especially in the parietal brain areas, in comparison to the other two groups
Wislowska et al. [89]	18 UWS 17 MCS 26 HC	slow waves and sleep spindles not statistically varied between day and night in patients changes in day and night in the power spectra as well as signal complexity evident in MCS but not in UWS diurnal fluctuations of the frequency power ratios associated with level of consciousness, via CRS-R CRS-R significantly positively correlated with density of sleep spindles during the night period in parietal areas negative correlation between amount of slow waves during the night period and the CRS-R
Rossi Sebastiano et al. [83]	49 UWS 36 MCS	signal attenuation as only EEG pattern during sleep time in around 1/3 of the UWS patients slow-wave sleep (but not REM) and non-REM 2 stages more often in MCS than in UWS presence of slow-wave sleep best tool to classify UWS and MCS duration of slow-wave sleep significantly correlated with the CRS-R
Zieleniewska et al. [90]	8 UWS 4 MCS− 2 MCS+ 5 EMCS	power of sleep spindles lower in UWS compared to MCS and EMCS detrended fluctuation analysis of the power profile of slow waves and spindles showed values normally over 1 for conscious patients calculated spectral entropy lower for UWS compared to other patient groups
Mertel et al. [82]	16 UWS 16 MCS 10 TC	behavioral and electrophysiological signs of sleep in all, expect for 1 UWS TC and MCS patients spent a significantly higher amount of time in sleep during nighttime than during daytime, not for UWS 12% of MCS and 44% of UWS, but 0 TC had no REM sleep 21% of MCS and 62% of UWS no sleep spindles for those with sleep spindles, the amplitude and number significantly lower comparing TC

**Table 3:** Overview of findings for diagnosis and evoked potentials; abbreviations can be found in Abbreviation.

Authors and Reference	Patient Sample	Finding
Schoenle and Witzke [94]	43 UWS 23 near UWS 45 non UWS	N400 UWS most likely no N400 could differentiate the groups
Kotchoubey et al. [95]	38 UWS 38 MCS 22 CS	cortical responses for all UWS patients with background activity higher than 4 Hz, but could not be found in patients with background activity lower than 4 Hz P300 more frequent P300 components correlated with lower level of disability N100 more frequent N100 components related to a lower level of disability N100 more frequent in MCS than in UWS P200 P200 more frequent in MCS compared to UWS
Perrin et al. [96]	5 UWS 6 MCS 4 LIS 5 HC	P300 P300 components as response to their own name in all LIS and MCS, as well as in 3 UWS patients comparing HC to MCS and UWS, delayed P300 in patients
Schnakers et al. [97]	8 UWS 14 MCS 12 HC	P300 passive and active (count own name) task: MCS, as well as HC, larger P300 to their own name (observed in passive and active condition) UWS patients no differences between active and passive condition
Qin et al. [98]	4 Coma 6 UWS 2 MCS	MMN present in 7 patients
Fischer et al. [99]	16 UWS 11 MCS	P300 novelty P300 responses in 7 patients, but overall no discrimination between MCS and UWS novelty P300 less frequent anoxia than other etiologies MMN MMN response in 5 patients, but overall no discrimination between MCS and UWS
Boly [100]	8 UWS 13 MCS 22 HC	MMN effective connectivity during MMN revealed impaired backward connectivity in UWS
Cavinato et al. [101]	6 UWS 11 MCS 10 HC	P300 MCS patients, similar to healthy controls, progressive increase in P300 latency in agreement with the level of complexity of the stimulus UWS no such modulation of P300 latency
Faugeras et al. [102]	22 UWS 19 MCS 8 CS 10 HC	MMN trend of relation between CRS and MMN presence of MMN not different between UWS and MCS, but less significant in UWS compared to MCS amplitude of MMN higher for higher levels of consciousness global effect HC have a large global effect on the global field power plots, no other statistically significant groups relationship between CRS and the presence of global effect
Balconi et al. [103]	10 UWS 8 MCS 20 HC	N400 found in fronto-central areas in UWS, MCS and HC
Chennu et al. [104]	9 UWS 12 MCS 8 HC	P300 1 UWS showed P300a and P300b
Risetti et al. [105]	8 UWS 3 MCS	P300 all patients except 1 novelty P300 under passive condition considering active condition (counting the new stimulus) novelty P300 increased and wider topographical distribution, when comparing to the passive condition, only in MCS but not in UWS amplitude of the novelty P300 was found to be correlated with the total CRS-R score and even more with the auditory sub-score MMN MMN in all UWS and MCS under passive oddball stimulation
Sitt et al. [59]	75 UWS 68 MCS 24 CS 14 HC	2/7 potentials significantly differentiate UWS and CS but none distinguish UWS from MCS P300 P300 moderate different between patient groups univariate statistics (electrode-by-electrode) of the P300 topography discriminates UWS from MCS MMN MMN discriminates UWS from CS as well as MCS but does not discriminate UWS from MCS
Wijnen et al. [106]	11 UWS 22 HC	Visual evoked potentials Visual evoked potentials were smaller in amplitude and longer in latencies when comparing UWS to HC
Balconi and Arangio [107]	7 UWS 11 MCS	N400 all patients higher N400 peak amplitude in the fronto-central regions as an answer to incongruous words, peak was delayed to incongruous stimuli in these cortical areas UWS patients delayed N400 in incongruous conditions compared to MCS correlation between the clinical scales (CNC and DRS) and the peak amplitude as well as latency
Hauger et al. [108]	11 MCS− 9 MCS+ 20 HC	P300 HC stronger P300 response when counting own name compared to listening to the pitch change for all groups higher response to the counting task, at an individual level
Li et al. [109]	2 Coma 6 UWS 5 MCS 17 HC	P300 two paradigms: the first was sine tone and subject’s own name and the second was derived name and subject’s own name all HC P300 in both paradigms with a longer latency and two peaks in the second paradigm all MCS patients P300 in the first and most of them in the second paradigm most UWS patients no P300
Rohaut et al. [110]	15 UWS 14 MCS 19 HC	N400 N400 in UWS, MCS and HC LPC LPC in just 6 HC, 5 MCS and 1 UWS
Schnakers et al. [111]	10 UWS 8 MCS− 8 MCS+ 14 HC	P300 5 MCS+, 3 MCS− and 1 UWS enhanced P300 amplitude when comparing active and passive condition patients’ responses widely distributed over fronto-parietal amplitude of the response for patients with covert cognition lower in fronto-central electrodes compared with HC, but no difference to MCS+
Beukema et al. [112]	8 UWS 8 MCS 17 HC	N400 cortical responses in all patients, some exceeded what was expected from behavioral assessment not different between UWS and MCS
Gibson et al. [113]	7 UWS 4 MCS 2 EMCS 18 HC	P300 8 patients P300a but none P300b patients with command following had event-related potentials of attentional orienting
Real et al. [114]	29 UWS 16 MCS 14 HC	P300 P300 lower in patients than in HC, no difference UWS to MCS
Erlbeck et al. [115]	13 UWS 3 MCS 3 EMCS	MMN MMN was identified in 2 patients N400 no response in most patients LPC LPC in 2 patients
Sergent et al. [116]	4 UWS 8 MCS 1 CS 15 HC	P300 9 HC significant P300 effect, also 1 UWS and 4 MCS, 0 CS most patients, who showed this effect, P300 latency to the own name paradigm temporally shifted Contingent negative variation significant in all HC and CS, 5 MCS and 3 UWS Action anticipation and attention shift to the cue side 8 HC, 0 CS, 1 MCS and 2 UWS Significant contextual modulation 3 HC, 1 CS, 1 MCS and 0 UWS Local incongruence detection 11 HC, 1 CS, 4 MCS and 1 UWS Global incongruence detection only the early part using source reconstruction, anterior cingulate cortex, caudal part, involved 8 HC, 1 MCS and 0 UWS Lateralized readiness potential 8 HC, 2 MCS but 0 UWS
Wang et al. [117]	6 UWS 5 MCS 5 HC	P300 increased P300 latency in UWS compared to other groups amplitude significantly different for UWS source of the P300 response located at the frontal lobe for the HC and at the temporal lobe for patient groups MMN higher MMN latency for UWS compared to other groups source of the MMN in frontal lobe for HC and in the temporal lobe for UWS and MCS
Kempny et al. [118]	5 UWS 11 MCS 12 HC	P300 statistically significantly different EEG responses comparing own name and another person’s name some response differences even similar to HC
Rivera-Lillo et al. [119]	10 UWS 3 MCS 10 HC	event-related synchronization across trials in the theta and delta bands patients lower number of trials with delta event-related synchronization a positive correlation between P300 and number of epochs with delta event-related synchronization was observed
Annen et al. [120]	15 UWS 23 MCS 2 EMCS 12 HC	P300 no different presence of P300 performance UWS compared to MCS or even to etiology (traumatic vs. non-traumatic) performances of 2 different stimuli (auditory and vibrotactile) independent from each other
Wu et al. [121]	20 UWS 22 MCS	P300 pronounced frontal P300 in MCS but not in UWS frontal P300 in non-traumatic patients clearer than in traumatic patients N100 N100 response in both MCS and UWS LPC no LPC in UWS

**Table 4:** Overview of findings for prognosis and resting-state EEG; abbreviations can be found in Abbreviation.

Authors and Reference	Patient Sample	Follow-Up	Finding
Schnakers et al. [44]	16 Coma 13 UWS 30 MCS 13 EMCS	12 months	Nonlinear measures patients who recovered higher bisprectral indices
Babiloni et al. [125]	50 UWS 30 HC	3 months	Spectral power alpha band: source power of occipital parts nearly null in not recovered patients, low in recovered patients and high in HC positive correlation between the recovery and the power of alpha source Patients evolving into an MCS: occipital alpha source power values between those values of patients recovering and not recovering from UWS
Fingelkurts et al. [126]	14 UWS 7 MCS	6 months	Spectral power variability and diversity of EEG in patients not surviving significantly lower than in patients who survived bad outcome associated with higher probability of slow theta and delta oscillations, in combination but also alone patients who survived higher probability of alpha and fast theta oscillations, in combination or alone
Sarà et al. [49]	23 UWS 40 HC	6 months	Nonlinear measures UWS patients who had the lowest approximate entropy values stayed UWS or died patients with high values of approximate entropy became MCS or even better
Fingelkurts et al. [127]	14 UWS	3 months	Functional connectivity strength as well as the number of functional connections was statistically higher in the first assessment (3 months post-injury) for patients who recovered compared to patients who did not recover Similar results alpha, beta 1 (from 15 to 25 Hz) and beta 2 (from 25 to 30 Hz) bands
Sitt et al. [59]	75 UWS 68 MCS 24 CS 14 HC	<42 days	Spectral power theta band: the higher the values of the normalized power, the higher the chance of recovery
Schorr et al. [63]	58 UWS 15 MCS 24 HC	12 months	Functional connectivity parietal and fronto-parietal coherence predict recovery from UWS to MCS delta and theta frequencies: the parietal coherence values significantly higher in the group which improved when compared to the group which did not improve coherence between frontal and parietal regions were higher in delta and theta but also alpha and beta frequencies coherence values of parietal delta and theta frequencies as well as fronto-parietal theta and alpha frequencies high, recovery of UWS predicted with a sensitivity of 73% and a specificity of 79%
Chennu et al. [128]	23 UWS 17 MCS− 49 MCS+ 11 EMCS 4 LIS 26 HC	12 months	Functional connectivity delta frequency network centrality predict outcome negative outcome (measured by GOS-E) for patients with strong connections of parietal and central areas positive outcome diminished delta connectivity Graph theory Non-traumatic patients positive outcome: significantly higher mesoscale modularity in delta band Traumatic patients significantly higher microscale clustering coefficients for networks of the delta frequency
Stefan et al. [66]	51 UWS 11 MCS	589.26 ± 1125.32 days	Spectral power power of alpha and delta frequencies performed even better at predicting outcome than indexing consciousness Functional connectivity coherence for all frequencies higher with improved outcome transfer entropy predicts outcome in the delta and alpha bands prognostic power: weighted symbolic mutual information in the alpha band Graph theory average clustering coefficient calculated from thresholding alpha and beta coherence prediction clustering coefficient in the theta range also significant path length no significant results Microstates microstate A most informative, i.e., duration of state in the delta band, the frequency and percentage time spent in this state in the theta band as well as the frequency of the microstate in the band from 2 to 20 Hz all significant Nonlinear measures approximate entropy in the alpha band successful prediction outcome but worse than permutation entropy in the delta and theta band
Bai et al. [34]	31 UWS 20 MCS 20 HC	3 months	Functional connectivity frontal quadratic phase self-coupling in the theta band significantly differentiates between patients who recover and those who do not
Bareham et al. [69]	16 UWS 15 MCS− 7 MCS+ 1 EMCS	3 months	Spectral power predict the CRS-R of the next measurement by the present EEG recordings
Kustermann et al. [129]	98 Coma	3 months	Graph theory lower clustering coefficient as well as higher path length variance and modularity for patients with a favorable outcome, at a group level variance in the path length best positive predictive value for favorable outcome as well as specificity for unfavorable outcome, above-chance values for negative predictive value and accuracy

**Table 5:** Overview of findings for prognosis and sleep patterns; abbreviations can be found in Abbreviation.

Authors and Reference	Patient Sample	Follow-Up	Finding
Oksenberg et al. [80]	11 UWS 6 HC	6 months	REM sleep characteristics but no significant differences between UWS who recovered and those who did not
Valente et al. [130]	24 Coma	12–34 months	better outcome via GOS significantly correlated with better polysomnography pattern with well-structured elements (REM and/or non-REM) appearance of organized sleep patterns predicted positive outcome, namely, full recovery or mild disability, with a sensitivity and specificity of 100% and 83%, respectively
Cologan et al. [84]	10 UWS 10 MCS	6 months	presence of sleep spindles related to clinical improvement
Mouthon et al. [88]	4 MCS 1 EMCS 5 CS 10 HC	1.5–16.1 months	parietal slow-wave activity build-up lowest in patients with poor outcome
Arnaldi et al. [131]	27 Coma	18.5 ± 9.9 months	better outcome correlated with visual index indication of sleep integrity, younger age and better clinical baseline sleep integrity best results, adding quantitative sleep index empowered prediction
Wislowska et al. [89]	18 UWS 17 MCS 26 HC	1–150 months	parietal sleep spindles linearly correlated with outcome
Yang et al. [132]	75 Coma	1 month	significant correlation between consciousness state after one month for patients in coma and the on-admission sleep EEG patterns higher modified Valente’s grade correlated with a higher likelihood of regaining consciousness

**Table 6:** Overview of findings for prognosis and evoked potentials; abbreviations can be found in Abbreviation.

Authors and Reference	Patient Sample	Follow-Up	Finding
Kotchoubey et al. [95]	38 UWS 38 MCS 22 CS	6 months	MMN MMN related to better outcome
Fischer et al. [133]	50 Coma	3 months	P300 P300 presence highly correlated with recovery of coma comparing MMN and P300: P300 higher specificity and sensitivity all patients, except 1, who showed parietal component in the late part of P300 woke up
Qin et al. [98]	4 Coma 6 UWS 2 MCS	3 months	MMN presence of MMN correlated with recovery of consciousness
Cavinato et al. [134]	34 UWS	12 months	P300 detectable P300 more often in patients who regained consciousness compared to those who did not
Faugeras et al. [138]	22 UWS	3–4 days	Bekinschtein protocol [137] 2 UWS showed neural signature of consciousness by the given protocol clinical signs of consciousness after 3 to 4 day
Faugeras et al. [102]	22 UWS 19 MCS 8 CS 10 HC	3–4 days	Global effect only UWS patients showing global effect improved consciousness
Xu et al. [139]	58 UWS	1 year	somatosensory evoked potentials correlated with outcome
Steppacher et al. [135]	53 UWS 39 MCS	2–14 years	P300 P300 in many UWS and MCS patients but not correlated with outcome N400 significant relationship between N400 occurrence and recovery
Wijnen et al. [106]	11 UWS 22 HC	2–3 years	Visual stimuli visual evoked potentials from the first measurement were related to the long-term outcome
Li et al. [109]	2 Coma 6 UWS 5 MCS 17 HC	1, 2, 3, 6, 9, and 12 months	P300 patients with a two-peak P300 to the oddball own name paradigm: higher chance of awakening within short time
Estraneo et al. [136]	71 UWS 76 MCS	6 months	P300 no correlation with outcome and EEG background activity or P300 to event-related potentials

**Table 7:** Overview of different values and their correlation with consciousness; abbreviations can be found in Abbreviation. d is Cohen’s d and the values in parenthesis are the confidence intervals. Fz, Cz, Pz, Oz refer to the EEG electrodes’ location. Papers that do not present enough data to calculate Cohen’s d are not included in the table.

Value	Ref	Comparison	Comment	Cohen’s d	Confidence Interval
alpha power	[52]	MCS vs. UWS	Frontal	0.60	(−0.19, 1.40)
			Posterior	0.85	(0.04, 1.66)
			Left hemisphere	0.70	(−0.10, 1.50)
			Right hemisphere	1.00	(0.18, 1.82)
	[55]	HC vs. MCS		1.50	(0.50, 2.50)
		HC vs. UWS		1.79	(0.71, 2.88)
	[56]	HC vs. DOC		2.64	(1.92, 3.36)
	[59]	CS vs. UWS		1.47	(1.19, 1.81)
		MCS vs. UWS		0.82	(0.66, 1.00)
	[62]	MCS vs. UWS	Fz	2.81	(0.98, 4.65)
			Cz	2.31	(0.65, 3.97)
			Pz	1.83	(0.31, 3.35)
	[66]	MCS vs. UWS		0.14	(0.04, 0.25)
approximate entropy	[12]	HC, CS vs. MCS	Eyes closed	1.71	(0.99, 2.43)
			Auditory, Verbal	1.49	(0.80, 2.19)
			Auditory, Music	1.96	(1.22, 2.71)
		HC, CS vs. UWS	Eyes closed	3.50	(2.60, 4.4)
			Auditory, Visual	2.70	(1.92, 3.48)
			Auditory, Music	3.23	(2.37, 4.09)
		MCS vs. UWS	Eyes closed	2.1	(1.27, 2.93)
			Auditory, Verbal	1.41	(0.67, 2.16)
			Auditory, Music	1.33	(0.59, 2.07)
	[49]	HC vs. DOC		2.83	(2.19, 3.47)
	[66]	MCS vs. UWS		0.25	(0.07, 0.43)
average clustering coefficient	[67]	MCS vs. UWS		−1.00	(−1.39, −0.61)
characteristic path length	[66]	MCS vs. UWS	Alpha	0.54	(0.40, 0.70)
			Beta	0.54	(0.36, 0.74)
clustering coefficient	[56]	HC vs. DOC		1.27	(0.7, 1.84)
	[66]	MCS vs. UWS		0.51	(0.47, 0.54)
	[68]	HC vs. MCS+	Delta	−1.08	(−1.28, −0.89)
			Theta	−0.98	(−1.17, −0.79)
		HC vs. MCS−	Delta	−1.69	(−1.99, −1.39)
			Theta	−1.61	(−1.90, −1.31)
		HC vs. UWS	Delta	−1.03	(−1.41, −0.66)
			Theta	−1.06	(−1.43, −0.68)
coherence	[66]	MCS vs. UWS	Alpha	0.51	(0.36, 0.7)
			Beta	0.40	(0.32, 0.47)
delta power	[52]	MCS vs. UWS	Frontal	−0.77	(−1.58, 0.03)
			Posterior	−0.97	(−1.79, −0.15)
			Left	−0.77	(−1.58, 0.03)
			Right	−0.93	(−1.75, −0.12)
	[55]	HC vs. UWS	Pz	−1.21	(−2.2, −0.23)
			Oz	−1.34	(−2.34, −0.33)
	[56]	HC vs. DOC		−2.63	(−3.35, −1,.91)
	[59]	CS vs. UWS		−1.24	(−1.47, −1.04)
		MCS vs. UWS		−0.70	(−0.87, −0.54)
	[62]	MCS vs. UWS	Fz	−2.99	(−4.94, −1.09)
			Cz	−2.61	(−4.38, −0.85)
			Pz	−2.52	(−4.25, −0.79)
	[66]	MCS vs. UWS		−0.29	(−0.54, −0.04)
dynamic functional connectivity	[65]	MCS vs. UWS	Alpha spectral connectivity	0.84	(0.1, 1.59)
			Gamma spectral connectivity	0.99	(0.23, 1.75)
entropy	[48]	HC vs. MCS		1.06	(0.38, 1.74)
		HC vs. UWS		2.02	(1.22, 2.81)
		HC vs. Coma		3.85	(2.28, 5.42)
		MCS vs. UWS		1.18	(0.56, 1.79)
		MCS vs. Coma		1.83	(0.8, 2.86)
		UWS vs. Coma		0.36	(−0.57, 1.29)
global effect	[59]	CS vs. UWS		1.24	(1.11, 1.37)
		MCS vs. UWS		0.43	(0.37, 0.49)
imaginary part coherence	[52]	MCS vs. UWS	Inter-hemisphere delta	−0.55	(−1.34, 0.24)
			Inter-hemisphere theta	0.35	(−0.43, 1.13)
			Inter-hemisphere alpha	0.83	(0.02, 1.64)
			Frontal to posterior delta	0.85	(0.04, 1.66)
			Frontal to posterior theta	1.10	(0.27, 1.93)
			Frontal to Posterior alpha	0.59	(−0.20, 1.38)
			Left delta	0.64	(−0.16, 1.43)
			Left theta	0.73	(−0.07, 1.53)
			Left alpha	0.71	(−0.09, 1.51)
			Right delta	0.50	(−0.29, 1.29)
			Right theta	0.50	(−0.29, 1.29)
			Right alpha	0.32	(−0.46, 1.10)
Kolmogorov–Chitain complexity	[59]	CS vs. MCS	Mean	0.87	(0.62, 1.14)
			Fluctuation	−0.47	(−0.7, −0.25)
		CS vs. UWS	Mean	1.29	(1.00, 1.67)
			Fluctuation	−0.62	(−0.87, −0.4)
		MCS vs. UWS	Mean	0.43	(0.25, 0.62)
			Fluctuation	−0.14	(−0.32, 0.04)
LPC	[110]	MCS vs. UWS	Presence	1.13	(−0.23, 3.29)
Lempel–Ziv complexity	[12]	HC, CS vs. MCS	Eyes closed	2.59	(1.76, 3.4)
			Auditory, Verbal	1.48	(0.79, 2.18)
			Auditory, Music	1.54	(0.84, 2.25)
		HC, CS vs. UWS	Eyes closed	4.17	(3.16, 5.18)
			Auditory, Verbal	2.84	(2.04, 3.65)
			Auditory, Music	2.48	(1.73, 3.23)
		MCS vs. UWS	Eyes closed	2.00	(1.18, 2.82)
			Auditory, Verbal	1.75	(0.96, 2.54)
			Auditory, Music	1.26	(0.52, 1.99)
local-community paradigm correlation	[67]	MCS vs. UWS		−0.954	(−1.34, −0.57)
local efficiency	[67]	MCS vs. UWS		−1.19	(−1.60, −0.78)
microstates	[51]	HC vs. MCS	Total number of ms	5.34	(2.49, 8.20)
			Posterior delta	−15.86	(−23.58, −8.14)
			Posterior theta	−19.96	(−29.63, −10.30)
			Posterior slow alpha	−3.22	(−5.20, −1.23)
			Posterior fast alpha	29.93	(15.50, 44.35)
			Anterior delta	−5.41	(−8.30, −2.52)
			Anterior theta	−8.73	(−13.11, −4.36)
			Anterior slow alpha	−0.56	(−1.85, 0.72)
			Anterior fast alpha	10.70	(5.41, 15.99)
		HC vs. UWS	Total number of ms	7.22	(4.43, 10.00)
			Posterior delta	−19.78	(−26.89, −12.67)
			Posterior theta	−12.56	(−17.16, −7.97)
			Posterior slow alpha	−5.89	(−8.25, −3.54)
			Posterior fast alpha	40.72	(26.21, 55.22)
			Anterior delta	−6.16	(−8.60, −3.72)
			Anterior theta	−9.33	(−12.820, −5.85)
			Anterior slow alpha	−1.83	(−3.09, −0.57)
			Anterior fast alpha	13.95	(8.88, 19.02)
		MCS vs. UWS	Total number of ms	−1.19	(−2.23, −0.16)
			Posterior delta	−2.72	(−4.04, −1.40)
			Posterior theta	−0.52	(−1.48, 0.45)
			Posterior slow alpha	−3.00	(−4.36, −1.63)
			Posterior fast alpha	8.54	(5.53, 11.55)
			Anterior delta	−0.05	(−1.00, 0.90)
			Anterior theta	−0.62	(−1.59, 0.36)
			Anterior slow alpha	−0.46	(−1.43, 0.51)
N100	[95]	MCS vs. UWS		0.48	(0.37, 0.59)
	[96]	HC vs. LIS	Latency	−0.35	(−1.68, 0.97)
		HC vs. MCS	Latency	−2.67	(−4.30, −1.04)
		HC vs. UWS	Latency	−1.78	(−3.24, 0.31)
		LIS vs. MCS	Latency	−2.13	(−3.71, −0.56)
		LIS vs. UWS	Latency	−1.53	(−3.02, −0.04)
		MCS vs. UWS	Latency	−0.51	(−0.69, 1.70)
	[101]	HC vs. MCS	Sine tone	−0.77	(−1.82, 0.27)
			SON	−0.48	(−1.51, 0.55)
			OFN	−0.77	(−1.82, 0.28)
		HC vs. UWS	Sine tone	−1.85	(−2.87, −0.83)
			SON	0.059	(−0.80, 0.91)
			OFN	0.07	(−0.78, 0.93)
		MCS vs. UWS	Sine tone	−0.92	(−0.12, −1.90)
			SON	0.51	(−0.50, 1.52)
			OFN	0.61	(−0.41, 1.62)
N200	[96]	HC vs. LIS	Latency	0.44	(−0.88, 1.78)
		HC vs. MCS	Latency	−3.60	(−5.52, −1.69)
		HC vs. UWS	Latency	−6.31	(−9.34, −3.28)
		LIS vs. MCS	Latency	−4.18	(−6.41, −1.96)
		LIS vs. UWS	Latency	−7.84	(−11.71, −3.99)
		MCS vs. UWS	Latency	−1.61	(−0.248, −2.98)
	[101]	HC vs. MCS	Sine tone	0.19	(−0.83, 1.20)
			SON	−0.25	(−1.26, 0.77)
			OFN	0.55	(−0.48, 1.58)
		HC vs. UWS	Sine tone	−0.88	(−1.78, 0.02)
			SON	0.16	(−0.70, 1.02)
			OFN	0.34	(−0.52, 1.20)
		MCS vs. UWS	Sine tone	−0.71	(−1.74, 0.31)
			SON	0.51	(−0.50, 1.52)
			OFN	−0.21	(−1.21, 0.79)
N400	[94]	no UWS vs. near UWS	Presence	0.54	(−0.33, 1.42)
		no UWS vs. UWS	Presence	1.47	(0.84, 2.22)
		near UWS vs. UWS	Pressence	0.93	(0.24, 1.71)
	[107]	MCS vs. UWS	Amplitude, congruous fronto-central	0.09	(−0.91, 1.10)
			Amplitude, incongruous fronto-central	−0.08	(−0.93, 1.08)
			Amplitude, congruous temporo-parietal	−0.15	(−1.15, 0.86)
			Amplitude, incongruous temporo-parietal	−0.07	(−1.08, 0.94)
			Amplitude, congruous occipital	0.17	(−0.83, 1.18)
			Amplitude, incongruous occipital	−0.01	(−1.03, 0.98)
			Latency, congruous fronto-central	−4.88	(−6.89, −2.87)
			Latency, incongruous fronto-central	−26.83	(−36.45, −17.21)
			Latency, congruous temporo-parietal	−12.55	(−17.14, −7.97)
			Latency, inconcgruous temporo-parietal	−10.45	(−14.32, −6.59)
			Latency, congruous occipital	−8.21	(−11.3, −5.12)
			Latency, incongruou occipital	−10.14	(−13.9, −6.39)
P200	[95]	MCS vs. UWS		0.48	(0.37, 0.59)
	[96]	HC vs. LIS	Latency	1.90	(0.32, 3.48)
		HC vs. MCS	Latency	−2.11	(−3.59, −0.635)
		HC vs. UWS	Latency	−3.87	(−6.10, −1.83)
		LIS vs. MCS	Latency	−3.49	(−5.47, −1.50)
		LIS vs. UWS	Latency	−5.52	(−8.39, −2.65)
		MCS vs. UWS	Latency	−1.55	(−0.20, −2.91)
	[101]	HC vs. MCS	Sine tone	0.24	(−0.77, 1.26)
			SON	0.15	(−0.87, 1.16)
			OFN	0.16	(−0.85, 1.18)
		HC vs. UWS	Sine tone	0.57	(−0.31, 1.44)
			SON	0.25	(−0.83,0.88)
			OFN	0.00	(−0.86, 0.86)
		MCS vs. UWS	Sine tone	0.23	(−0.77, 1.23)
			SON	−0.08	(−1.07, 0.92)
			OFN	−0.96	(−2.01, 0.8)
P300	[95]	MCS vs. UWS		0.46	(0.35, 0.56)
	[96]	HC vs. LIS	Latency	−1.64	(−3.16, −0.12)
		HC vs. MCS	Latency	−5.16	(−7.62, −2.70)
		HC vs. UWS	Latency	−8.76	(−12.79, −4.73)
		LIS vs. MCS	Latency	−3.22	(−5.12, −1.33)
		LIS vs. UWS	Latency	−5.31	(−8.10. −2.53)
		MCS vs. UWS	Latency	−1.04	(−2.31, 0.22)
	[101]	HC vs. MCS	Sine tone	−0.38	(−1.40, 0.64)
			SON	−0.72	(−1.76, 0.32)
			OFN	−0.50	(−1.53, 0.53)
		HC vs. UWS	Sine tone	0.28	(−0.58, 1.14)
			SON	0.11	(−0.75, 0.96)
			OFN	0.49	(−0.38, 1.36)
		MCS vs. UWS	Sine tone	1.40	(0.30, 2.50)
			SON	0.98	(−0.149, 1.93)
			OFN	1.07	(0.02, 2.13)
	[116]	HC vs. MCS	Occurance SON	0.35	(−0.04, 0.74)
		HC vs. UWS	Occurance SON	0.99	(0.23, 1.75)
		MCS vs. UWS	Occurance SON	0.64	(−0.24, 1.52)
	[117]	HC vs. MCS	Test run 1 Cz latency SON	−0.08	(−1.32, 1.16)
			Test run 1 Cz amplitude SON	0.13	(−1.11, 1.37)
			Test run 1 Cz latency OFN	−0.56	(−1.83, 0.70)
			Test run 1 Cz amplitude OFN	0.47	(−0.79, 1.73)
		HC vs. UWS	Test run 1 Cz latency SON	−1.88	(−3.30, −0.45)
			Test run 1 Cz amplitude SON	0.21	(−0.99, 1.40)
			Test run 1 Cz latency OFN	−0.41	(−1.6, 0.79)
			Test run 1 Cz amplitude OFN	0.61	(−0.61, 1.82)
		MCS vs. UWS	Test run 1 Cz latency SON	−1.81	(−3.22, −0.40)
			Test run 1 Cz amplitude SON	0.08	(−1.11, 1.26)
			Test run 1 Cz latency OFN	0.43	(−0.77, 1.63)
			Test run 1 Cz amplitude OFN	0.09	(−1.10, 1.27)
permutation entropy	[59]	CS vs. MCS	Theta mean	0.54	(0.25, 0.82)
			Alpha mean	0.74	(0.47, 1.04)
			Beta mean	0.51	(0.25, 0.78)
			Gamma mean	0.43	(0.18, 0.7)
			Theta fluctuation	−0.5	(0.7, −0.25)
			Alpha fluctuation	−0.54	(−0.78, −0.32)
			Beta fluctuation	−0.54	(−0.78, −0.32)
			Gamma fluctuation	−0.54	(−0.78, −0.32)
		CS vs. UWS	Theta mean	1.35	(1.09, 1.66)
			Alpha mean	0.95	(0.70, 1.24)
			Beta mean	0.36	(0.11, 0.62)
			Gamma mean	0.29	(0.04, 0.54)
			Theta fluctuation	−1.14	(−1.41, −0.91)
			Alpha fluctuation	−1.00	(−1.24, −0.78)
			Beta fluctuation	−0.43	(−0.66, −0.21)
			Gamma fluctuation	−0.32	(−0.54, −0.11)
		MCS vs. UWS	Theta mean	0.82	(0.66, 1.00)
			Alpha mean	0.40	(0.21, 0.58)
			Beta mean	−0.11	(−0.29, 0.07)
			Gamma mean	−0.11	(−0.29, −0.07)
			Theta fluctuation	−0.70	(−0.87, −0.54)
			Alpha fluctuation	−0.54	(−0.74, −0.36)
			Beta fluctuation	0.11	(−0.07, 0.29)
			Gamma fluctuation	0.18	(0.00, 0.36)
	[66]	MCS vs. UWS	Alpha	0.40	(0.32, 0.47)
phase lag index	[52]	MCS vs. UWS	Inter-hemisphere delta	−0.65	(−1.44, 0.15)
			Inter-hemisphere theta	0.00	(−0.78, 0.78)
			Inter-hemisphere alpha	1.30	(0.45, 2.15)
			Frontal to posterior delta	0.47	(−0.32, 1.25)
			Frontal to posterior theta	0.80	(−0.01, 1.61)
			Frontal to posterior alpha	0.39	(−0.39, 1.18)
			Left delta	0.00	(−0.78, 0.78)
			Left theta	0.84	(0.03, 1.65)
			Left alpha	0.70	(−0.1, 1.5)
			Right delta	0.03	(−0.75, 0.81)
			Right theta	0.37	(−0.42, 1.15)
			Right alpha	0.42	(−0.36, 1.21)
phase locking index	[59]	CS vs. MCS	Mean, delta	−0.07	(−0.32, 0.18)
			Fluctatuion, delta	−0.11	(−0.36, 0.14)
		CS vs. UWS	Mean, delta	−0.47	(−0.7, −0.25)
			Fluctatuion, delta	−0.54	(−0.78, −0.32)
		MCS vs. UWS	Mean, delta	−0.43	(−0.62, −0.25)
			Fluctatuion, delta	−0.43	(−0.62, −0.25)
quadratic self-coupling	[34]	HC vs. MCS	Alpha	0.46	(−0.12, 1.04)
		HC vs. UWS	Alpha	1.02	(0.34, 1.70)
		MCS vs. UWS	Alpha	0.40	(−0.18, 0.98)
quadratic self-coupling	[34]	HC vs. MCS	Theta	1.67	(1.01, 2.33)
		HC vs. UWS	Theta	2.07	(1.28, 2.87)
		MCS vs. UWS	Theta	0.91	(0.30, 1.51)
REM	[80]	HC vs. DOC	Duration	1.64	(0.41, 2.87)
	[81]	MCS vs. UWS	Presence	Inf	(0.17, Inf)
	[82]	HC vs. UWS	Time in REM	1.92	(1.50, 2.34)
		MCS vs. UWS	Time in REM	0.76	(0.53, 0.99)
sleep spindels	[82]	Hc vs. MCS		Inf	(−0.25, Inf)
		HC vs. UWS		Inf	(0.48, Inf)
		MCS vs. UWS		0.69	(−0.2, 1.66)
	[84]	MCS vs. UWS		0.934	(0.45, 1.42)
	[85]	MCS vs. UWS		1.10	(0.10, 2.27)
slow-wave sleep	[81]	MCS vs. UWS	% power of waking vs. sleep (MCS) and eyes open vs. closed (UWS)	6.33	(2.78, 9.87)
			Presence	Inf	(0.58, Inf)
	[84]	MCS vs. UWS	Presence	1.16	(−0.07, 2.67)
small-worldness omega	[67]	MCS vs. UWS		1.24	(0.83, 1.65)
small-worldness omega efficiency	[67]	MCS vs. UWS		1.09	(0.69, 1.49)
spectral entropy	[62]	MCS vs. UWS	Mean Fz	2.51	(0.78, 4.24)
			Mean Cz	1.97	(0.41, 3.53)
			Mean Pz	1.86	(0.33, 3.39)
			Sd Fz	2.42	(0.72, 4.12)
			Sd Cz	1.89	(0.35, 3.43)
			Sd Pz	1.53	(0.08, 2.97)
			Cov Fz	2.32	(0.65, 3.99)
			Cov Cz	1.62	(0.16, 3.08)
			Cov Pz	1.26	(−012, 2.63)
theta power	[59]	CS vs. MCS	Normalized	0.14	(0.03, 0.25)
		CS vs. UWS	Normalized	0.70	(0.59, 0.82)
		MCS vs. UWS	Normalized	0.51	(0.45, 0.56)
	[62]	MCS vs. UWS	Fz	1.87	(0.51, 3.22)
			Cz	2.38	(0.9, 3.86)
			Pz	2.12	(0.70, 3.53)
transfer entropy	[66]	MCS vs. UWS	Alpha	0.62	(0.51, 0.74)
weighted symbolic mutual information	[54]	MCS vs. UWS	Anoxia	1.59	(1.18, 2.00)
			Traumatic	1.09	(0.89, 1.29)
			Stroke	0.82	(0.58, 1.06)
	[59]	CS vs. UWS	Theta	1.09	(0.97, 1.21)
		MCS vs. UWS	Theta	0.91	(0.85, 0.97)
	[66]	MCS vs. UWS	Theta	0.358	(0.13, 0.58)
			Delta	0.701	(0.47, 0.93)
			Alpha	0.213	(−0.01, 0.44)

**Table 8:** Overview of different values and the correlation with better outcome; abbreviations can be found in Abbreviation. d is Cohen’s d and the values in parenthesis are the confidence intervals. Papers that do not present enough data to calculate Cohen’s d are not included in the table.

Value	Ref	Comment	Cohen’s d	Confidence Interval
alpha power	[66]		0.51	(0.22, 0.79)
	[125]	Occipital	5.40	(4.41, 6.39)
approximate entropy	[66]		0.62	(0.33, 0.91)
bispectral index	[44]		0.73	(0.51, 0.95)
clustering coefficient	[66]	Beta	1.30	(0.97, 1.62)
		Alpha	1.30	(0.97, 1.62)
		Theta	0.83	(0.53, 1.13)
	[129]		−0.88	(−0.97, −0.79)
coherence	[63]	Partial, theta	0.95	(0.29, 2.09)
		Partial, delta	0.87	(0.25, 1.74)
		fronto-parietal, alpha	0.78	(0.14, 1.74)
		fronto-parietal, theta	0.87	(0.25, 1.81)
	[66]	Theta	1.09	(0.78, 1.40)
		Alpha	0.43	(0.15, 0.71)
		Beta	0.62	(0.33, 0.91)
delta power	[66]		−0.66	(−0.37, −0.95)
global effect	[102]		Inf	(−0.01, Inf)
imaginary part of coherence	[66]	Beta	0.95	(0.65, 1.26)
mesoscale modularity	[128]	Delta, non-traumatic	1.08	(0.73, 1.43)
microscale clustering coefficient	[128]	Delta, traumatic	1.09	(0.71, 1.48)
microstate A	[66]	Duration, delta	0.95	(0.65, 1.26)
		Frequency, theta	0.95	(0.65, 1.26)
		Time in A, theta	1.47	(1.13, 1.80)
		Frequency, 2–20Hz	0.87	(0.57, 1.17)
MMN	[95]		0.76	(0.56, 0.95)
	[98]		Inf	(0.29, Inf)
modified Valente’s grade	[132]		0.45	(0.12, 0.78)
modularity	[129]		0.61	(0.52, 0.70)
N400	[135]	Wavelet	0.91	(0.79, 1.03)
		Human	2.15	(1.99, 2.30)
organized sleep patterns	[130]		1.31	(0.91, 1.72)
P300	[109]		Inf	(0.11, Inf)
	[134]		2.20	(1.19, 3.29)
	[135]	Wavelet	0.25	(0.15, 0.36)
		Human	0.44	(0.33, 0.55)
permutation entropy	[66]	Delta	0.78	(0.49, 1.08)
		Theta	1.35	(1.02, 1.68)
	[89]		1.00	(0.25, 1.75)
quadratic phase self-coupling	[34]	Theta, frontal	−0.84	(−1.63, −0.05)
sleep spindles	[84]		Inf	(0.89, Inf)
	[89]	Density, MCS/MCS+ vs. death	1.13	(0.50, 1.76)
		Density, UWS/SD- vs. death	0.96	(0.35, 1.57)
somatosensory evoked potentials	[139]		1.74	(1.18, 3.03)
theta normalized power	[59]		0.78	(0.51, 1.09)
transfer entropy	[66]	Delta	0.74	(0.45, 1.03)
		Alpha	1.09	(0.78, 1.40)
variance of path length	[129]		0.75	(0.66, 0.84)
weighted symbolic mutual information	[66]	Alpha, 32s	0.87	(0.57, 1.17)
		Alpha, 8s	0.78	(0.49, 1.08)
		Delta, 8s	0.70	(0.41, 0.99)

## Abbreviation

**Abbreviation**	**Meaning**
AUC	Area Under the Curve
CNC	Coma/Near Coma
CRS	Coma Recovery Scale
CRS-R	Coma Recovery Scale-Revised
CS	Conscious Subject
DOC	Disorders of Consciousness
DRS	Disability Rating Scale
EEG	Electroencephalogram
EMCS	Emerging Minimally Conscious State
fMRI	Functional Magnetic Resonance Imaging
GCS	Glasgow Coma Scale
GOS	Glasgow Outcome Scale
GOS-E	Glasgow Outcome Scale-Extended
GLS	Glasgow–Liège Scale
HC	Healthy Control
ICS	Innsbruck Coma Sale
LIS	Locked-In Syndrome
LPC	Late Positive Component
MCS	Minimally Conscious State
MCS+	Minimally Conscious State Plus
MCS−	Minimally Conscious State Minus
MMN	Mismatch Negativity
Ms	Milliseconds
OFN	Other First Name
OR	Odds Ratio
PET	Positron Emission Tomography
QEEG	Quantitative Electroencephalogram
REM	Rapid Eye Movement
Sd	Standard Deviation
SND	Severe Neurocognitive Disorder
SON	Subject’s Own Name
TC	Tetraplegic Controls
TMS	Transcranial Magnetic Stimulation
UWS	Unresponsive Wakefulness Syndrome
WHIM	Wessex Head Injury Matrix
